# Integrating physical activity into the workplace: opportunities for enhancing employee health

**DOI:** 10.3389/fpsyg.2026.1812858

**Published:** 2026-06-17

**Authors:** Gabriele Signorini, Raffaele Scurati, Andrea Bosio, Angelo Pagano, Emanuele Magaldi, Pietro Luigi Invernizzi, Marta Rigon

**Affiliations:** 1 Department of Biomedical Sciences for Health, Università degli Studi di Milano, Milan, Italy; 2 Human Performance Laboratory, Mapei Sport Research Centre, Olgiate Olona, Italy

**Keywords:** desk workers, musculoskeletal disorders, physical well-being, psychological well-being, sedentary behavior

## Abstract

**Introduction:**

Human health is closely linked to physical, social, and ecological environments. Since people spend a large portion of their time at work, this setting plays a key role in well-being. Desk workers often remain sedentary for 8–10 h daily, increasing the risk of musculoskeletal disorders and pain. The UP150 office concept aims to promote movement during working hours, supporting the mind–body connection, improving relationships, and enhancing both health and work quality. This study builds on previous research by evaluating the effects of the UP150 concept after 2 years of implementation.

**Methods:**

A focus group identified key intervention strategies, and 41 employees from an architectural company were recruited. Twenty-four participants were assigned to the experimental group and 17 to the control group. The experimental group followed the UP150 model for 2 years, while the control group maintained usual routines. Every 6 months, measures included motor efficiency, physical activity, sedentary behavior, psychological well-being, and socio-psychological job aspects. At the end, semi-structured interviews explored participants’ experiences with UP150.

**Results:**

The focus group showed strong organizational commitment but highlighted the need to improve the social environment and reduce sedentary time. Over 2 years, the experimental group improved motor efficiency, particularly in cardiorespiratory fitness and flexibility (shoulder and back mobility), while the control group showed no significant changes. The experimental group also reported increased light and moderate physical activity and better psychological well-being compared to controls.

**Discussion:**

The UP150 model offers a practical framework to reduce sedentary behavior and promote a healthier workplace culture. Integrating structured movement into daily routines helps employees break prolonged sitting and adopt more active habits, supporting both physical and psychological health. Overall, its adoption can provide sustainable, long-term benefits for individuals and organizations.

## 1 Introduction

The World Health Organization (WHO) has consistently highlighted the connection between population health and the physical, social, and ecological environments. Understanding the relationship between the mind, body, and environment is essential for determining the appropriate levels of physical activity necessary to improve or maintain health. The WHO’s “One Health” approach expands this understanding, encouraging a holistic perspective on health challenges such as the reduction of NCDs rather than relying on reductionist models often used in clinical practice (World Health Organization, 2022; Winkler et al., 2025; Zinsstag et al., 2011).

**Figure 1:**
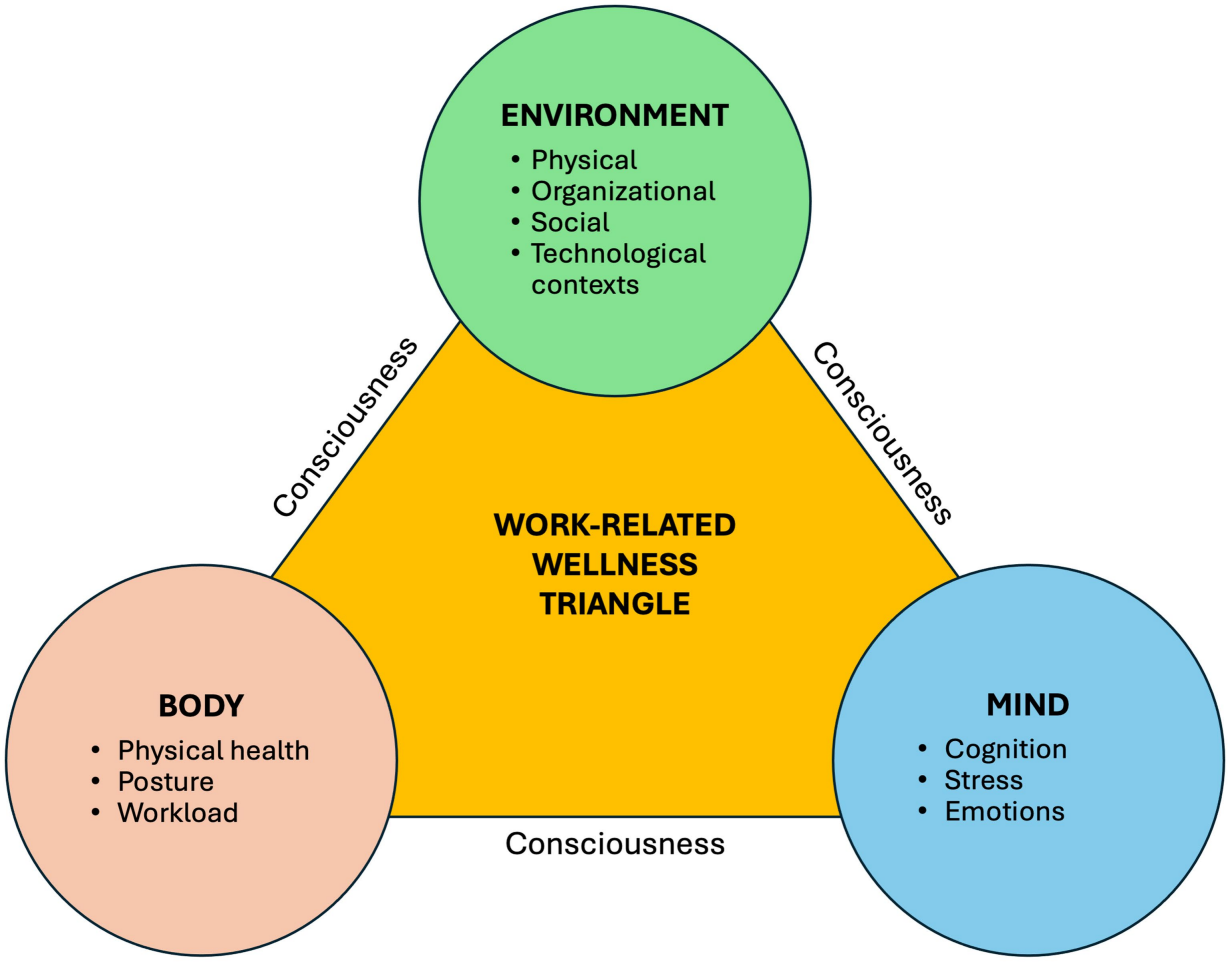
Representation of the work-related wellness triangle.

The *Work-related Wellness Triangle* (Figure 1) is an original conceptual framework designed to illustrate the interactions among the subjective, psychological, and social dimensions of workplace well-being. While not historically established in occupational psychology, it builds on multidimensional models of work well-being and aligns with the holistic perspective described by Facco and Tagliagambe (Facco and Tagliagambe, 2020), which emphasizes the interdependence of environment, body, and mind—echoing Hippocrates and early Pre-Socratics holistic thoughts (Kaufman, 2018; Perez-Padron et al., 2010), a vision that resonates with contemporary approaches to complexity, towards which current scientific research is increasingly moving (Popa et al., 2015). In this framework, the environment is positioned as the enabling context for both body and mind, reinforcing the need for workplaces that actively support employee health and balance.

**Figure 2:**
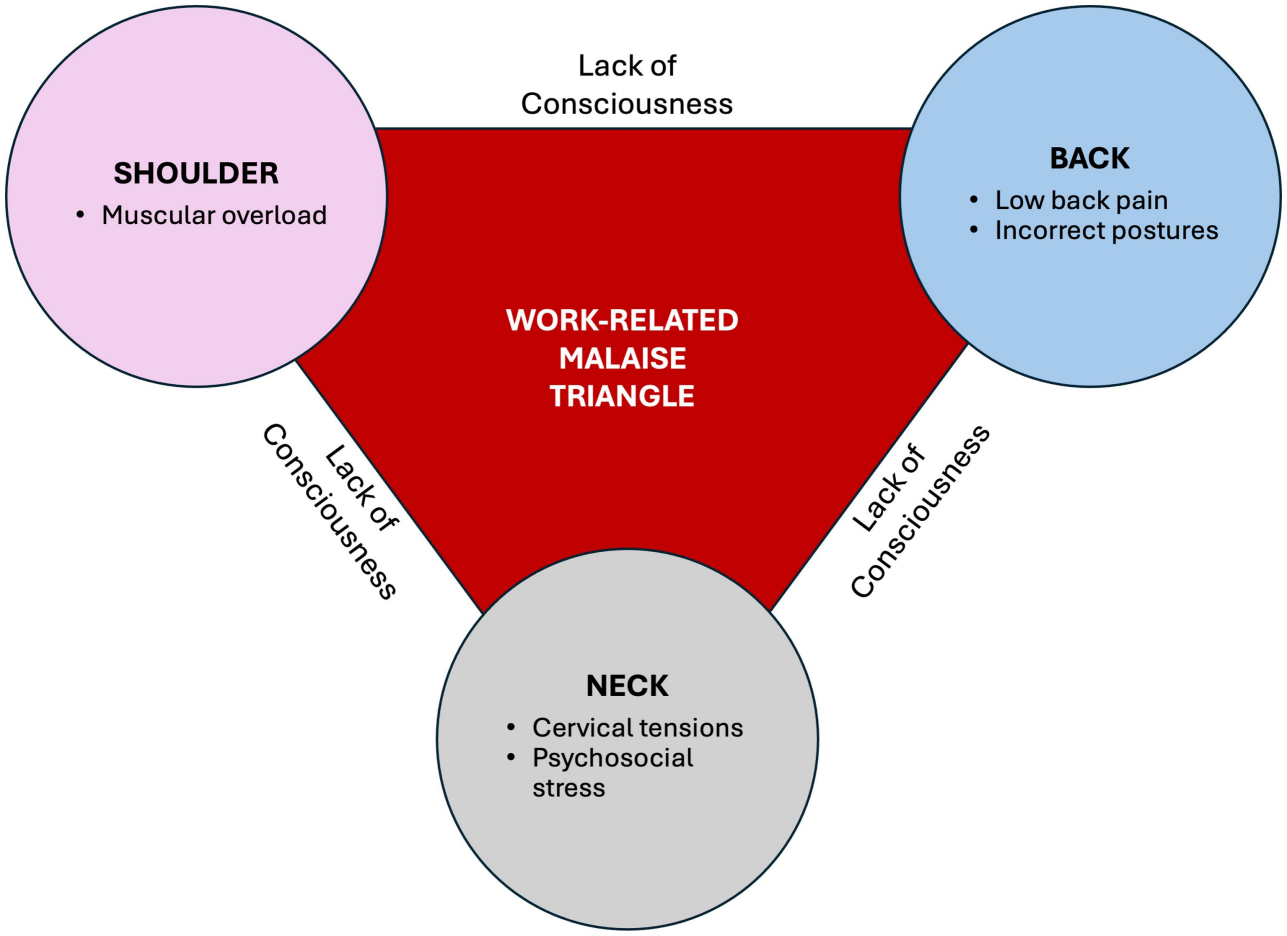
Representation of the work-related malaise triangle.

Complementing this, the *Work Malaise Triangle* (Figure 2) represents the frequent co-occurrence of neck, shoulder, and lower back discomfort, linking these musculoskeletal problems to physical, postural, and psychosocial stressors. Like the *Wellness Triangle*, it is an original heuristic construct illustrating how imbalances across body, mind, and environment can manifest as pain or dysfunction. Together, the two triangles provide a conceptual tool to integrate ergonomic, psychological, and environmental factors in workplace health interventions (Chandwani et al., 2019; Russeng et al., 2021; Wang et al., 2021).

This integrated approach aligns with ISO 45001, the international standard for occupational health and safety management systems, which emphasizes systematic risk identification and mitigation. It also supports Environmental, Social, and Governance (ESG) criteria, particularly regarding employee well-being, fair labor practices, and responsible governance (Butlewski and Broda, 2025; Widanarko et al., 2014). By guiding organizations to protect human capital and promote responsible practices, this model contributes to the United Nations Sustainable Development Goals (SDGs): SDG 3 (Good Health and Well-Being), emphasizing prevention of occupational illnesses and promotion of physical and mental health; SDG 8 (Decent Work and Economic Growth), focusing on safe, fair, and productive working conditions; and SDG 12 (Responsible Consumption and Production), highlighting sustainable and ethical organizational practices (Shrivastava and Shrivastava, 2019). Together, these goals connect workplace health strategies to broader social, economic, and environmental outcomes.

Significant progress has been made in understanding how workplace environments affect employee well-being (Widanarko et al., 2014). However, there has been less focus on how these environments—comprising physical, social, cultural, and technological factors—interact to influence health outcomes (Mattke et al., 2013; McCoy et al., 2014). In general population, the dose–response relationship between sedentary time and all-cause mortality shows a gradual increase beginning at around 7.5 h per day, with the risk becoming more pronounced for sedentary times exceeding 9.5 h per day. Sitting for 10 h per day is associated with a 48% increased risk of all-cause mortality compared to sitting for 7.5 h per day (Ekelund et al., 2019). Sedentary behavior also poses a significant risk for the development of musculoskeletal disorders and pain, with prevalence rates ranging from 33.8 to 95.3% (Demissie et al., 2024). Despite growing awareness of the negative effects of sedentary behavior, research on effective systemic interventions remains limited (Kavaratzis and Choi, 2026). Recent studies indicate that desk workers, especially those working remotely, are particularly at risk, experiencing high rates of back pain, migraines, shoulder tension, and neck pain—issues that are often worsened by inadequate home office setups (Mohammadian et al., 2025). These findings highlight the urgent need for systemic changes in workplace environments to mitigate the adverse health impacts of sedentary behavior.

The transition from the *Work-related Malaise Triangle* to the health-promoting *Work-related Wellness Triangle* necessitates a significant change in organizational culture. This transformation is not simply about managing symptoms reactively; instead, it requires the proactive design of a work ecosystem that incorporates physical activity and well-being into daily practices. This shift is acknowledged in the ISO 45001 standards and aligns with ESG-focused governance models. Engaging in physical activity has been shown to reduce chronic musculoskeletal pain, lower cardiovascular risks, and alleviate mental health issues, thereby enhancing overall employee well-being (Ji, 2021).

Although workplace physical activity interventions have demonstrated short-term benefits, maintaining long-term engagement and sustainable health improvements remains a challenge. Previous programs—such as structured exercise sessions, standing desks, walking meetings, or company-provided on-site gyms accessible during work breaks—have improved physical activity and fitness but often lack systematic integration into daily work routines (Dugdill et al., 2008). The UP150 project (Ufficio Proattivo 150) addresses these gaps by embedding physical activity into the workplace through active break stations, a mobile app, and wellness coaching, creating a continuous system of engagement, feedback, and improvement (Invernizzi et al., 2022). Unlike prior interventions, UP150 combines environmental, behavioral, and organizational components to foster lasting employee participation.

Building on earlier studies (Invernizzi et al., 2022; Signorini et al., 2024; Signorini et al., 2022), the present research evaluates the long-term effectiveness of UP150 over 2 years, examining physical fitness, mental health, job-related stress, and employees’ perceptions of the work environment. This study aims to clarify how workplace well-being initiatives can sustain physical activity, reduce stress, and promote overall health, hypothesizing significant improvements in fitness, mental health, and long-term engagement among employees. Specifically, this study will assess physical fitness, mental health, and job-related stress during the intervention. Additionally, it will explore employees’ perceptions of the work environment and their experiences with the UP150 initiative.

This research aims to provide valuable insights into how incorporating physical activity into the workplace can enhance physical fitness, reduce job-related stress, and improve mental health over time. Additionally, it will provide evidence on the sustainability of employee engagement in physical activity, clarifying how workplace well-being initiatives can lead to long-term health benefits. It is hypothesized that integrating the UP150 model into the workplace will result in significant improvements in employees’ physical fitness, reductions in job-related stress, and enhanced mental health over 2 years. Furthermore, this initiative is expected to positively influence employees’ long-term engagement in physical activity, leading to sustainable health improvements and overall well-being.

## 2 Materials and methods

### 2.1 Participants

Employees from an Italian architectural company (Progetto CMR) were recruited to participate in this study. This study represents the second year of an intervention trial, the first year of which was previously reported in a mid-term study by Signorini et al. (2024). On that occasion, the study sample size was determined through an *a priori* power analysis using G*Power (version 3.1.9.4, Universität Kiel, Germany), based on the F-test family (ANOVA, repeated measures, within–between interaction). An effect size f of 0.25 and a statistical power of 0.95 were assumed, resulting in a minimum required sample size of 32 participants. In the same phase, the call for participation was sent by email to employees, with the employer’s agreement, and participants were selected according to the following criteria: performing office-based work, working at least 6 h per day, being in good health, and having no conditions that limit participation in physical activity.

Therefore, the participants in the present study (*n* = 41) are the same as those included in the study by Signorini et al., who continued into the second year of the trial, and allocated as follows: experimental group (EG; *n* = 24, 7 females and 17 males; age 41.0 ± 12.08 years; height 1.75 ± 0.09 m; weight 71.97 ± 17.10 kg; BMI 23.14 ± 3.68 kg/m^2^) and control group (CG; *n* = 17, 7 females and 10 males; age 42.12 ± 10.31 years; height 1.71 ± 0.10 m; weight 68.07 ± 14.89 kg; BMI 23.03 ± 3.47 kg/m^2^). Four participants (CG, *n* = 3; EG, *n* = 1) who dropped out at the end of the first year of the trial were excluded from the present analyses.

This study was approved by the Ethic Committee of the University of Milan (approval nr. 84/20).

### 2.2 Procedures

Before the two-year experimental trial, a focus group was conducted to assess employees’ views of the company and their needs. Subsequently, CG participants continued their everyday work tasks for 2 years (from October 2022 to June 2024), while the EG experienced the UP150 concept for the same period. During these 2 years, both groups were periodically tested on motor efficiency, weekly physical activity, psychological well-being, and social and psychosocial perceptions of their jobs. We considered the normal working year, starting in October and ending at the end of June, to avoid the Italian summer holidays, when employees usually work fewer days. Hence, all considered variables were evaluated at the beginning and end of this working period, yielding a total of 4 measures (one at the beginning and one at the end of each working year). At the end of the two-year trial, a semi-structured interview was conducted to evaluate employees’ perceptions of the UP150 concept.

Over the course of the two-year trial, the UP150 concept successfully integrated well-being, physical exercise, and technology into the workflow. This approach applied principles from self-determination theory (Deci and Ryan, 2000) as well as concepts related to the extended mind, which have been discussed in previous research (Invernizzi et al., 2022; Signorini et al., 2024; Signorini et al., 2022; Invernizzi et al., 2021). Table 1 summarizes the main rationale and key aspects of the UP150 concept.

**Table 1:** Summary of the UP150 (wellness, movement, and technology within the workflow) concept.

Aspect	Rationale
Project goal	Integrate physical exercise into the workflow to improve well-being, performance, and the corporate environment.
Context	Transformation of contemporary organizational work models; Centrality of the employee well-being in work performance; Overcoming the separation between work and physical activity.
Self-representation and change	The employee develops a new capacity for self-representation; Unconventional working identity is built; The body becomes an active part of the working process; The body becomes an instrument of awareness and self-transformation.
Cognitive and emotional niches	Corporate physical practices generate cognitive and emotional niches; Physical and mental spaces that influence attention, motivation, and the quality of relationships; Cognitive-emotional niches modify the way a company thinks about its activities from within.
Role of technology	Technological tools as mediators of change; Support for monitoring, integration of motor practices and workflow continuity; Technology does not replace the body but enhances it.
*Extended mind* and work	Building an *extended mind* by integrating body/brain, technology, and work environment; New thinking emerges from the interaction between the individual and the context.

The employees of EG were educated and monitored in the activity by wellness coaches (professional figures with degrees in sports sciences) who promoted the appropriate use of all active break stations, the technological devices integrated into the office, and the dedicated UP150 app, using the principles of need-supportive communication. Wellness coaches helped and guided workers in performing physical activities at work, based on trial testing and analysis of each employee’s specific needs. Moreover, they planned and managed exercises and the UP150 experience, motivating them to pursue adequate physical activity (at light and moderate intensities) and a healthy routine. The wellness coaches were present in the company 2 days a week and were available remotely (by email or text) during working days. A complete description of the wellness coach’s figure is provided in a previously published study (Signorini et al., 2024).

### 2.3 Measures

#### 2.3.1 Focus group

Before the experimental trial began, 19 participants (9 females, 10 males) were enrolled in an initial focus group. The group was comprised of representative workers from any of the following areas: (a) project managers; (b) business unit; (c) marketing and communications; (d) graphic and design; (e) accounting and financial planning; (f) office design. The focus group was convened to understand the company’s situation regarding employee psychophysical wellness and work-related health needs. Recruited workers have varying levels of experience in the company, ranging from 1 to 10 years. Five were aged 25–35, five 36–45, five 46–55, and four 56+. During the focus group, four stimuli were proposed to participants. The first one was oriented to the vision of one’s own working reality: *“If you have to use an image describing your company, which one would you choose?”* The second stimulus aimed to investigate the degree of wellness in working environments: *“Highlight a specific event during your workday/week in which you felt good or comfortable, and an event where you felt uncomfortable.”* The third stimulus wanted to highlight the employees’ needs regarding working wellness: *“How could your company concretely change to improve your working wellness?”* The last stimulus aimed to investigate the employees’ movement needs: *“What are your needs related to physical exercise? Do you feel like you move enough, or would you like to do more?”* Each focus group was audio recorded, transcribed and analyzed using the line-by-line coding of the grounded theory methodology (Corbin and Strauss, 1990).

#### 2.3.2 Motor efficiency

Motor efficiency was assessed using the Cubo Fitness Test, a submaximal test based on perceived exertion, helpful in evaluating participants’ cardiovascular, muscular, and flexibility fitness. It had already been validated and used in this type of population; moreover, the same test was used to assess the participant’s motor efficiency during the first year of the trial, as previously reported in the literature (Invernizzi et al., 2022; Signorini et al., 2024; Invernizzi et al., 2021). The Cubo Fitness Test comprehend 5 submaximal tests based on internal load: (1) Ruffier test based on heart rate (Cardiorespiratory fitness: scores from 20 to 0; lower values represent higher performances); (2) 30 s push-up based on Borg CR-10 scale (Muscular fitness: scores from 0 to 10; higher values represent higher performances; moderate perceived exertion) gives a scores normalized for age and sex final score; (3) 30 s seated sit-up based Borg CR-10 scale (Muscular fitness: scores from 0 to 10; higher values represent higher performances; moderate perceived exertion) gives a scores normalized for age and sex final score; (4) shoulder mobility based on stretching intensity scale (Flexibility fitness: the result are expressed in cm, the higher the result the worst is the performance; maximal stretching without pain); (5) seated seat and reach based on stretching intensity scale (Flexibility fitness: the results are expressed in cm, the higher the result the better is the performance; maximal stretching without pain). The test gives a final total score named “index of motor efficiency,” representing the general motor efficiency (score ranged from 0 to 100, normalized for age and gender; the higher the score, the better the performance). The details of the test and its reliability were explained in previous studies (Invernizzi et al., 2022; Signorini et al., 2024; Invernizzi et al., 2021).

#### 2.3.3 Weekly physical activity

To measure participants’ weekly physical activity and sedentary behavior, Axivity AX3 triaxial accelerometers (Axivity Ltd., Newcastle upon Tyne, UK, 2013) have been used (Doherty et al., 2017). During the four main assessments (October 2022, June 2023, October 2023 and June 2024), participants wore accelerometers on the non-dominant hand wrist for a whole week, from 5:00 p.m. on Monday to 8:00 a.m. the following Monday (Dieu et al., 2017). The accelerometers captured acceleration over a range of *±*16 g at 100 Hz (Arvidsson et al., 2019). The raw triaxial data were exported using OmGUI software version 1.24 (Axivity Ltd., Newcastle upon Tyne, UK, 2013). The results are reported in weekly minutes of activity at light, moderate, and vigorous intensity. The cut-offs used by the OmGUI software for determining the intensity are: Light = 1.5 < and < 3.99 MET; Moderate = 4.0 < and < 6.99 MET; Vigorous = more than 7.0 MET (Axivity, 2015). Accelerometers measured the comprehensive participants’ physical activity across the entire week, which includes activities within and outside the work environment.

#### 2.3.4 Psychological well-being

Psychological well-being was evaluated using the Psychological General Well-being Index. Consists of 22 items rated on a 6-point Likert scale that investigate: anxiety, depression, positive mood, self-control, general health, and vitality. The total of all items is used to create an overall general well-being index. The Italian version of the PGWBI was employed in the present study (Grossi et al., 2006).

#### 2.3.5 Job social and psychological perceptions

In the present study, the Job Content Questionnaire was selected to assess the social and psychological characteristics of employees’ jobs (Karasek et al., 1998; Karasek and Theorell, 1990). We used the adapted and validated Italian version, consisting of 49 questions based on a 4- to 5-point Likert scale and some open questions (Baldasseroni et al., 2001). The questionnaire evaluates three main job characteristics: decision latitude, job demands, and social support, as explained in previous research (Signorini et al., 2024).

#### 2.3.6 Final interview

A final semi-structured interview was proposed to participants at the end of the experimental period to investigate their perceptions of the entire UP150 concept and its features. Moreover, the interview aimed at investigating the possible repercussions on company and personal wellness. The interviews were delivered in person by a researcher not involved in the other phases of intervention. The entire interview was recorded using a digital recorder and subsequently transcribed for the analysis. The questions proposed were:

- At the end of the UP150 project, do you perceive any difference in your health status?
- Which of the features of the UP150 project is more useful, and which one is less? Why? Can the project be improved? If yes, how?
- What do you think about the training system based on consciousness and effort perception?
- Do you think the UP150 project has had any effect on you or your work context? If yes, how?
- Do you have any suggestions for developing the concept within your company or in other companies?

The employees’ answers were analyzed using the grounded theory methodology as explained in the “focus group” section.

### 2.4 Statistical analysis

The normality of the data was verified using the Kolmogorov–Smirnov test and by evaluating the Skewness and Kurtosis. To evaluate the effectiveness of the UP150 project, a mixed repeated ANOVA (4×2; Time x Group) was performed for all considered parameters (Cubo Fitness Test, Accelerometer measures, Psychological General Well-being Index, and Job Content Questionnaire). Homogeneity and sphericity were assessed; if sphericity was violated, the Greenhouse–Geisser correction was applied. To evaluate differences between groups and time points, a *post hoc* test with the Bonferroni correction was used. The alpha level was set at 0.05. Effect sizes were evaluated using partial eta squared (η^2^_p_). The η^2^_p_ cut-off considered were the following: Small effect = 0.01, Medium effect = 0.06, Large effect = 0.14 or higher. Concerning post-hoc tests, Cohen’s d was assessed and following cut-offs were used: d = 0.2 (small effect), d = 0.5 (medium effect), d = 0.8 (large effect). The level of significance was set at *α* = 0.05 (*p* < 0.05). Due to the long duration of the trial, some missing data occurred; therefore, the pairwise deletion method was applied.

### 2.5 Qualitative analysis

A qualitative analysis was conducted using grounded theory methodology. This involved transcribing and analyzing recorded interviews in a series of phases. The first phase entailed open coding, during which we extracted pre-labels (main themes) from the transcriptions. Following this, we identified and grouped pre-labels with similar themes into labels. Next, these labels were coded into categories, which were quantified based on their frequency of appearance in the focus groups and interviews. Following Corbin and Strauss (1990), categories appearing in less than 50% of the responses were considered “sporadic,” those in the 50–70% range were labeled “typical,” and categories exceeding 71% were deemed “general.” The category with the highest frequency was identified as the core category. We then established connections between categories to better explain the phenomena described in the focus groups and interviews. Two evaluators carried out the grounded theory process, which was subsequently presented and discussed with the research team.

## 3 Results

**Figure 3:**
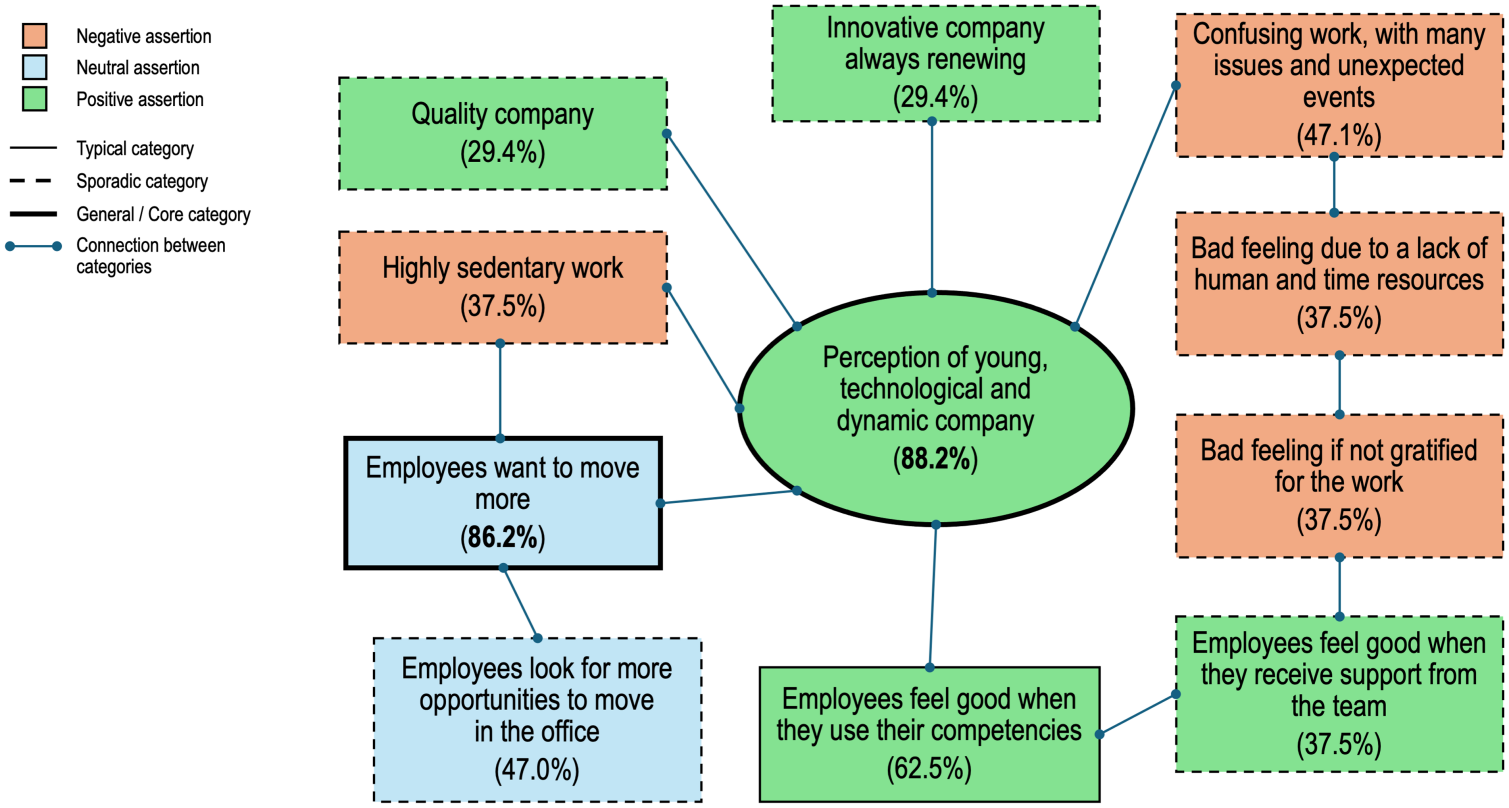
Employees’ perceptions of the work environment as resulting from the focus group carried out before starting the UP150 intervention.

The focus group outlined an innovative, young, and dynamic work environment where employees feel gratified when valued and used for their competencies. Moreover, employees felt better when they perceived support from their colleagues. Nevertheless, workers reported difficulty achieving their work goals due to a perceived lack of time and human resources. Furthermore, employees perceived themselves as too sedentary and sought more opportunities for movement. Figure 3 displays the most representative results from the focus groups.

The data were normally distributed and homogeneous.

**Figure 4:**
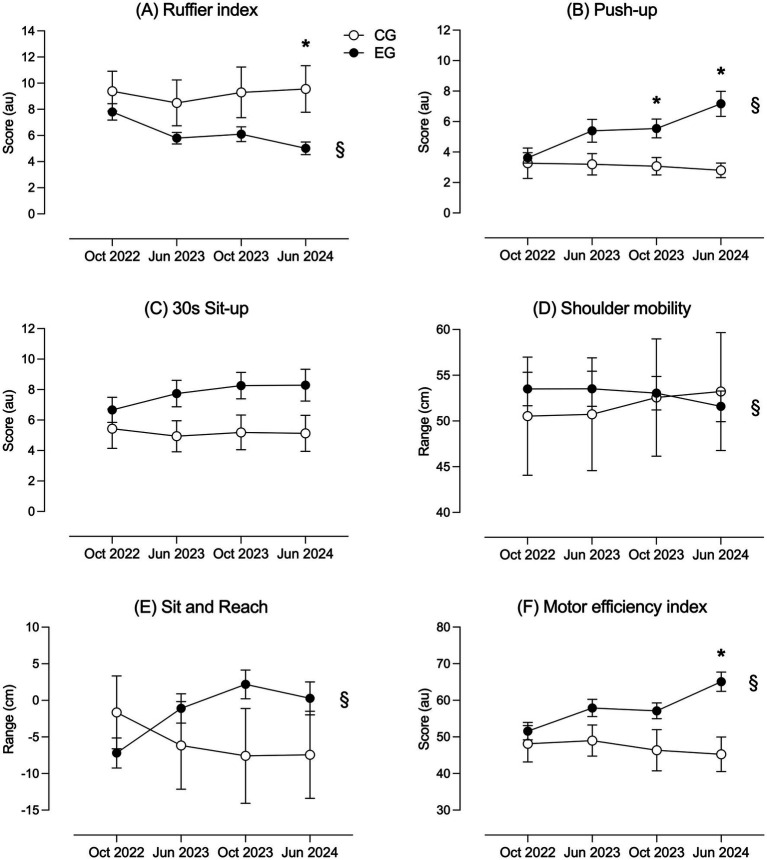
Motor efficiency measured by the Cubo Fitness test: comparison between the control group (CG) and the group of employees participating in the UP150 intervention (EG). Panels show the following motor efficiency variables: **(A)** Ruffier index, **(B)** Push-up, **(C)** 30s Sit-up, **(D)** Shoulder mobility, **(E)** Sit and Reach, and **(F)** Motor efficiency index. *Significant difference between EG and CG (*p* < 0.05); §Significant interaction time × group (*p* < 0.05).

The EG and CG resulted similarly at the very beginning of the trial (Ruffier test: d = 0.357, *p* = 0.628; 30 s push-up test: d = 0.949, *p* = 1.000; 30 s seated sit-up test: d = 0.376, *p* = 1.000; shoulder mobility test: d = −0.335, *p* = 1.000; seated sit and reach test: d = 0.511, *p* = 1.000; index of motor efficiency: d = 0.364, *p* = 1.000). The motor efficiency measured by the Cubo Fitness Test reported significant changes in the Ruffier test (Interaction time*group: *p* = 0.004; η^2^_p_ = 0.121), Thirty seconds push-up test (Interaction time*group: *p* < 0.001; η^2^_p_ = 0.326), Shoulder mobility test (Interaction time*group: *p* = 0.002; η^2^_p_ = 0.139), Sit and Reach test (Interaction time*group: *p* < 0.001; η^2^_p_ = 0.394; Figure 4). In all these sub-tests, the EG reported positive changes, indicating better results than CG. Similarly, the index of motor efficiency reported significant changes in favor of EG (Interaction time*group: *p* < 0.001; η^2^_p_ = 0.224). No interaction or effects were detected in the 30-s seated sit-up test (Interaction time*group: *p* = 0.127; η^2^_p_ = 0.054).

**Figure 5:**
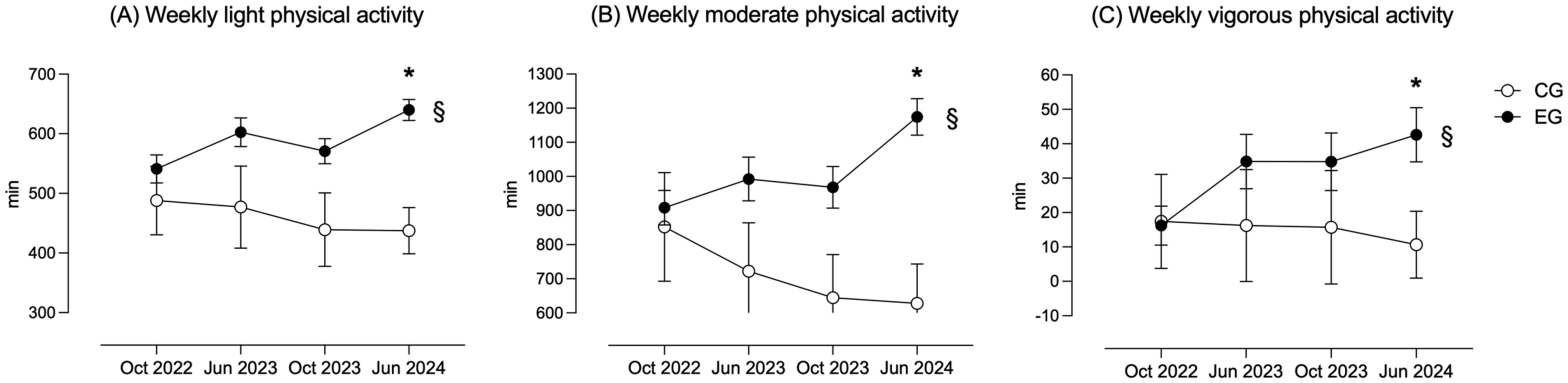
Weekly physical activity: comparison between the control group (CG) and the group of employees participating in the UP150 intervention (EG). Panels show weekly physical activity at different intensity levels: **(A)** light, **(B)** moderate, and **(C)** vigorous. *Significant difference between EG and CG (*p* < 0.05); §Significant interaction time*group (*p* < 0.05).

Concerning weekly physical activity, EG and CG did not show a difference at the beginning of the trial (light: d = 0.084, *p* = 1.000; moderate: d = 0.138, *p* = 1.000; vigorous: d = 0.027, *p* = 1.000). EG reported an increase of light (interaction time*group: *p* = 0.002; η^2^_p_ = 0.158) and moderate weekly physical activity (interaction time*group: *p* < 0.001; η^2^_p_ = 0.226), showing better outcomes in EG (Figure 5). In the same way, small differences with a small effect size were observed between CG and EG in vigorous physical activity during the trial (interaction time*group: *p* = 0.029; η^2^_p_ = 0.0098).

Similar psychological well-being characterized the participants of the two groups at the beginning of the trial (positivity: d = 0.498, *p* = 1.000; general health: d = 0.964, *p* = 0.670; vitality: d = 0.806, *p* = 0.905; anxiety: d = 0.424, *p* = 1.000; depression: d = 0.272, *p* = 1.000; self-control: d = 0.114, *p* = 1.000; total score: d = 0.745, *p* = 1.000). The psychological well-being questionnaire reported significant interactions (time*group) for Positivity, General Health, Vitality, and total score (General well-being). In all the interactions, the experimental group reported increased and better outcomes compared to the control group. Results and significances are reported in Table 2.

**Table 2:** Psychological well-being: time*group interactions of the psychological general well-being index results.

Subscale	Group	Oct 2022	Jun 2023	Oct 2023	Jun 2024	Sig.	ES
Anxiety	CG	15.4 ± 5.2	16.0 ± 3.3	15.5 ± 4.6	16.3 ± 4.9	0.749	0.025
	EG	15.4 ± 5.0	17.2 ± 4.7	15.8 ± 4.8	15.1 ± 4.7		
Depression	CG	11.9 ± 3.2	12.8 ± 1.3	12.4 ± 2.6	12.6 ± 1.5	0.579	0.040
	EG	12.1 ± 3.5	13.4 ± 1.4	13.4 ± 1.6	13.4 ± 1.2		
Positivity	CG	12.8 ± 2.6	11.5 ± 1.8	10.8 ± 2.5	11.4 ± 1.8	< 0.001	0.607
	EG	12.1 ± 1.9	13.2 ± 2.0	15.2 ± 2.9	14.6 ± 2.9		
Self-control	CG	10.8 ± 2.5	10.8 ± 2.4	11.0 ± 2.9	10.6 ± 2.4	0.491	0.049
	EG	11.6 ± 2.5	11.9 ± 2.3	11.2 ± 2.4	11.4 ± 2.4		
General health	CG	11.8 ± 2.2	11.2 ± 2.3	8.6 ± 3.9	11.7 ± 1.8	0.003	0.250
	EG	11.1 ± 1.8	12.9 ± 1.1	12.6 ± 1.8	12.9 ± 1.9		
Vitality	CG	12.9 ± 3.0	11.5 ± 2.8	10.9 ± 3.9	11.8 ± 2.6	0.006	0.228
	EG	11.2 ± 2.6	12.9 ± 3.1	14.3 ± 3.0	14.1 ± 2.5		
Total score	CG	75.6 ± 13.8	73.8 ± 11.5	69.1 ± 15.8	74.4 ± 11.4	0.019	0.185
	EG	73.5 ± 12.6	81.6 ± 10.8	82.4 ± 13.4	81.5 ± 10.1		

CG, control group; EG, experimental group. Scores are expressed in AU (mean ± SD). ES, effect sizes expressed as η^2^ p values.

In JCQ, the two groups were similar at the beginning of the trial (Job demand: d = 0.212, *p* = 1.000; social support: d = 0.873, *p* = 1.000; decision latitude: d = 0.224, *p* = 1.000). The JCQ, measuring job social and psychological perceptions, reported a significant interaction (time*group) for Job Demand and Social support variables, whereas no interaction was observed for decision latitude. Results and significances are reported in Table 3.

**Table 3:** Job social and psychological perceptions: time*group interactions of the Job Content Questionnaire results.

Job characteristic	Group	Oct 2022	Jun 2023	Oct 2023	Jun 2024	Sig.	ES
Decision latitude	CG	73.9 ± 10.6	72.2 ± 6.0	58.9 ± 19.8	62.4 ± 13.7	0.971	0.002
	EG	77.5 ± 7.5	76.8 ± 8.1	61.2 ± 25.4	62.9 ± 23.0		
Job demand	CG	34.5 ± 2.4	34.4 ± 3.6	33.5 ± 5.1	32.4 ± 4.9	<0.001	0.315
	EG	36.1 ± 3.7	32.3 ± 6.0	21.0 ± 12.8	19.0 ± 10.0		
Social support	CG	23.1 ± 5.2	25.4 ± 4.8	20.2 ± 4.4	21.1 ± 5.0	<0.001	0.317
	EG	19.7 ± 5.9	23.7 ± 3.9	25.7 ± 3.3	26.9 ± 5.7		

CG, control group; EG, experimental group. Scores are expressed in AU (mean ± SD). ES, effect sizes expressed as η^2^ p values.

**Figure 6:**
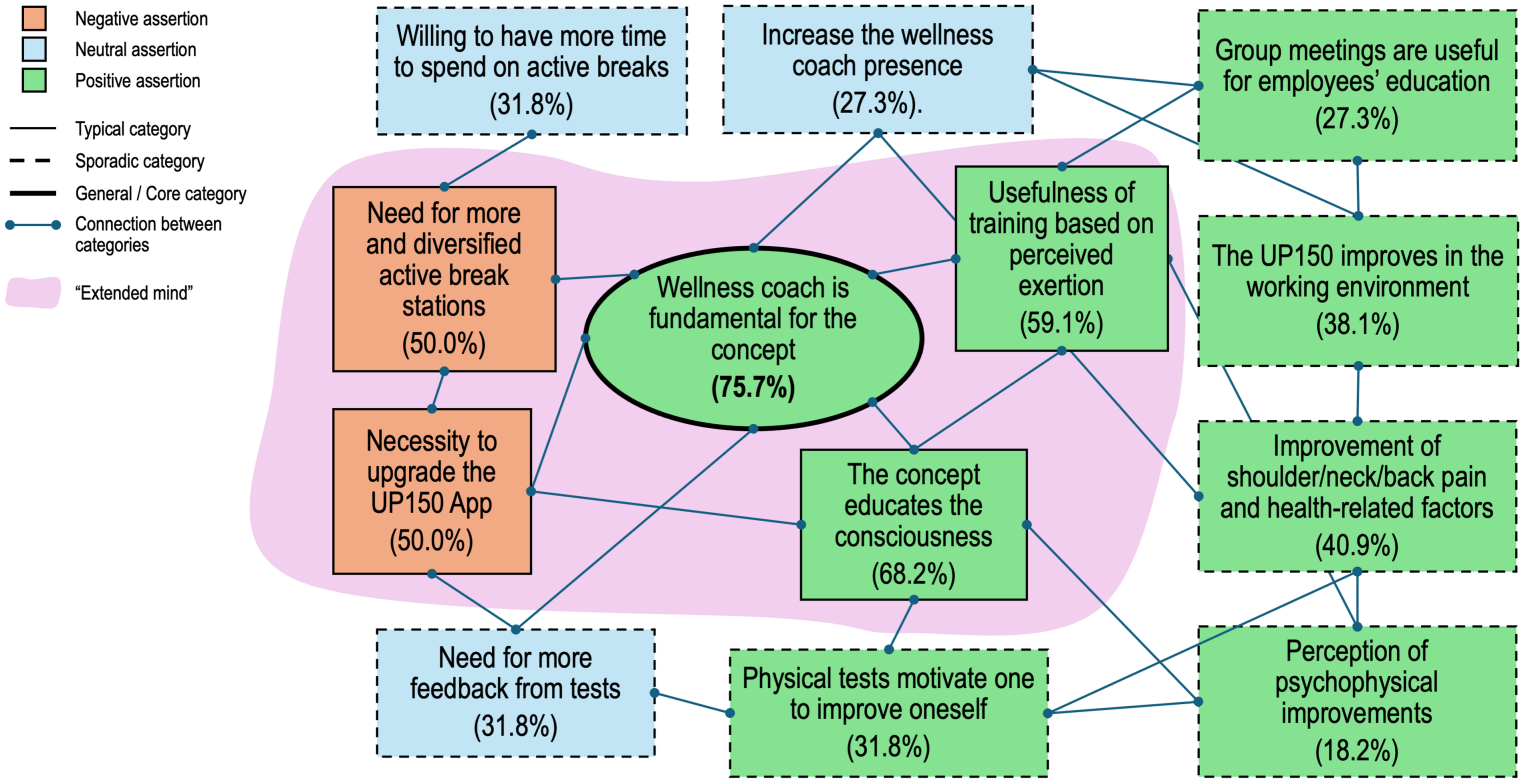
Employees’ perceptions of the work environment emerging from the focus group carried out after the UP150 intervention.

In the final interview, most participants (75%) reported the importance of wellness coaches for the UP150 concept. They reported the importance of education for consciousness (68.2%) and of a training method based on perceived exertion (59.1%). Nevertheless, some critical points emerged: participants reported the need for more diverse active break stations (50%) and an upgrade to the app (50%). Finally, participants reported a desire to spend more time on active breaks (31.8%), receive more feedback on tests (31.8%), and have a greater presence of wellness coaches at the company (27.3%). Results from the employees’ final interview based on the Grounded theory qualitative analysis are reported in Figure 6.

## 4 Discussion

This research builds on previous studies investigating the UP150 concept’s role in promoting workplace health, specifically focusing on its impact over 2 years of intervention. Participants in this study were 41 sedentary desk workers, employees of an Italian architectural company, who spent more than 6 h per day in sedentary behavior, which is typical for most desk workers (Stephens et al., 2018). Over the two-year trial, the experimental group exhibited significant improvements in motor efficiency, physical activity levels, psychological well-being, and job-related social perceptions. In contrast, the control group maintained stable levels across these variables.

Participants in the focus group initially described the company as “young and dynamic,” with an “open mind” toward new ideas. However, participants also reported challenges such as role ambiguity, resource shortages, and associated feelings of confusion and frustration. These issues were consistent with Vanishree’s findings (Vanishree, 2014), which noted that role ambiguity and work overload can negatively affect employee satisfaction and performance. Moreover, these results indicate a clear need for clarity, support, and autonomy within the workplace. In addition, employees described their work as predominantly sedentary and expressed a desire for increased physical activity, both during and outside working hours. These insights underscore the relevance of implementing a need-supportive communication approach within the UP150 intervention. According to Self-Determination Theory (Ryan & Deci, 2000), intrinsic motivation is fostered when individuals’ needs for autonomy, competence, and relatedness are supported. By employing need-supportive communication, the intervention can: (1) clarify the objectives and benefits of physical activity, reducing role ambiguity and uncertainty; (2) promote employees’ sense of personal responsibility for their health; (3) enhance competence by providing clear guidance and feedback on incorporating movement into the workday; and (4) strengthen relatedness by aligning activities with shared organizational values, such as collective well-being and the principles of One Health (World Health Organization, 2022; Winkler et al., 2025; Zinsstag et al., 2011). In summary, the focus group outcomes demonstrate that employees require both structural (Rossi et al., 2025) and psychological support to engage in workplace physical activity. Integrating need-supportive communication into the UP150 initiative not only addresses these needs but also fosters intrinsic motivation, ultimately enhancing the effectiveness of the intervention and contributing to employee well-being and engagement (Hallam et al., 2022).

During the trial, employees demonstrated a significant increase in motor efficiency, along with marked improvements in cardiovascular fitness and flexibility. Similar outcomes were noted in a previous one-year trial (Signorini et al., 2024). These findings confirm the retention of motor efficiency and indicate continued improvements over time. Most worksite physical activity interventions reported in the literature show high efficacy in the early months but are often abandoned by participants in the long term (Fletcher et al., 2008). The maintenance of improvements over time can represent a symptom of positive effect retention as indicated by the literature (Ryde et al., 2013). The enhancement in flexibility, especially back flexibility, is of particular interest as it correlates with reduced joint pain, a common issue for desk workers. Prolonged sitting and poor posture contribute to joint inflammation and muscle stiffness, as indicated by Mohammadian et al. (2025). Improvements in shoulder and back flexibility are likely to benefit neck and back health (Gobbo et al., 2019; Tunwattanapong et al., 2016). However, core strength did not show a significant change, as expected given the nature of the interventions. Prolonged sitting reduces abdominal muscle activation, and moderate-intensity physical exercise during work hours likely did not provide sufficient training volume for significant changes in strength (O'Sullivan et al., 2002; Spiering et al., 2021; Waongenngarm et al., 2015; Straker and Mathiassen, 2009). At the same time, the experimental group showed a consistent increase in weekly physical activity. The trend observed in the previous one-year trial continued, with light and moderate physical activities experiencing the most significant growth (Signorini et al., 2024).

The control group showed no significant changes in either motor efficiency or weekly physical activity outcomes, which highlights the effectiveness of the intervention in the experimental group. The observed improvements in motor efficiency and flexibility support previous studies suggesting that active breaks and ergonomic enhancements can alleviate musculoskeletal pain, such as neck, back, and shoulder discomfort, caused by prolonged sitting and poor posture (Gasibat et al., 2017; Walker et al., 2017) reducing the negative effects of the *work-related malaise triangle* mentioned in introduction section. The improvements in flexibility are particularly noteworthy, as they are directly related to reduced joint pain, a common issue among desk workers. These findings corroborate Pronk’s conclusions (Pronk, 2021), which highlighted the importance of ergonomically designed workstations and activity breaks in alleviating the negative effects of sedentary behavior. While core strength did not show significant improvement, this is a known limitation of sedentary workplace interventions. Achieving significant strength gains typically requires higher-intensity exercise and longer periods of activity, which are difficult to integrate into the typical workday (Spiering et al., 2021; Straker and Mathiassen, 2009). The increase in weekly physical activity, particularly in light and moderate-intensity activities, aligns with previous studies demonstrating that such interventions can lead to sustained behavior changes (Invernizzi et al., 2022; Signorini et al., 2022; Dishman et al., 2009; Parry et al., 2013). Notably, the vigorous physical activity directly measured using accelerometers can reasonably be attributed exclusively to activities performed outside the workplace for two main reasons. First, it would have been impractical and uncomfortable for employees to perform high-impact activities without appropriate sportswear, as they conducted activities at the workplace in standard work attire. Second, wellness coaches were present in the workplace to promote, supervise, and guide activities specifically at light-to-moderate intensity. The results in this study can be interpreted accordingly.

The intervention also positively impacted psychological well-being. Throughout the two-year trial, the experimental group experienced improvements in overall psychological well-being, especially in areas such as positivity, general health, and vitality. The connection between physical activity and psychological well-being is well-documented (Biddle et al., 2003). Numerous studies, particularly those conducted in office environments, have confirmed this relationship when physical activity interventions are implemented (Signorini et al., 2024; Signorini et al., 2022; Abdin et al., 2018; Lucini et al., 2021). We believe that this effect was influenced by our methodology, which included self-determination theory and need-supportive communication. These approaches promote feelings of relatedness, autonomy, and competence. Our hypothesis is further supported by the results from the Job Content Questionnaire (JCQ), which indicated an increase in perceived social support within the workplace. Social support is essential for achieving high levels of work wellness, as it fosters positive relationships and helps maintain motivation (Jolly et al., 2020). The JCQ also indicated a decrease in job demands, which are linked to the physical and psychological commitment required in work. The increased opportunity for active breaks likely contributed to a reduction in task pressure (Ghesmaty Sangachin et al., 2016; Rabal-Pelay et al., 2025).

The positive effects on psychological well-being observed in this study align with a substantial body of research that links physical activity to reduced stress and increased vitality (Kukić et al., 2023; Mahindru et al., 2023; Rozanski, 2023; Witard et al., 2016). The rise in positivity, general health, and vitality within the experimental group reinforces the connection between physical activity and emotional regulation. Engaging in regular physical activity improves mood and lessens anxiety, contributing to a healthier workplace environment (Pronk, 2014) and reducing fibromyalgia. The final interview reinforced these findings, describing an optimistic scenario in which the wellness coaches played a key role in the concept’s success. Employees appreciated the UP150 approach, particularly its emphasis on awareness and perceived exertion. This focus not only helped reduce joint pain (such as pain in the back, neck, and shoulders) but also improved workplace relationships suggesting a shift from the *work-related malaise triangle* to *work-related wellness triangle* framework (Widanarko et al., 2014). However, employees expressed a desire for more diverse active break stations, as the current options did not meet all their needs. The demand for additional active breaks led to the upcoming further development of the UP150 App, which enhances the use of active break stations and serves as an effective tool for promoting physical exercise, especially due to its motivational effects. The introduction of new active break stations and the UP150 App could further improve the overall concept, enhancing office ergonomics and fostering healthier movement habits. This integration promotes autonomous behaviors aimed at improving well-being, work performance, and self-awareness (Invernizzi et al., 2021; Commissaris et al., 2016; Jindo et al., 2019).

Follow-up results from the second year of intervention indicated minimal dropout, with only one participant in the experimental group withdrawing (the participant moved to another working position). Unlike other workplace interventions (Fletcher et al., 2008), the UP150 concept demonstrated high adherence and continued to yield positive effects. Interviews indicated that the positive effects of the program could not have been sustained without effective communication facilitated by wellness coaches and technology. Employees reported a positive experience with the program, with 76% of those interviewed expressing satisfaction. If the goal is to educate, it is essential to have skilled facilitators. The use of need-supportive communication was pivotal in achieving the desired outcomes. Employees recognized the continuous presence of wellness coaches, group educational sessions, and support—both in-person and remote—as valuable. By integrating physical activity into the workday, the UP150 project enables employees to redefine their personal and professional narratives. This approach fosters cognitive and emotional spaces (niches) that positively impact the corporate environment, prioritizing worker well-being as integral to professional performance. Technological tools play a crucial role in this process, enhancing the concept of the extended mind and encouraging physical practices that align with daily work activities (Clark, 2010; Clark and Chalmers, 1998). According to the extended mind concept, emotional processes are distributed across the body, technology (such as digital tools), and the environment (Clarkson et al., 2022; Mazeas et al., 2022; Table 1). The findings of this study reinforce the growing body of literature asserting that promoting physical activity in the workplace can contribute to better health outcomes and higher levels of physical literacy. This improvement is achieved through the development of healthy routines, proper education in movement, enhanced self-perception, and fostering autonomy in physical development (Commissaris et al., 2016; Jindo et al., 2019; Laranjo et al., 2021; Ryan and Deci, 2000).

A parallelism between the quantitative results and the interview findings reinforces the effectiveness of the UP150 program in promoting lasting improvements in both physical health and psychological well-being (Table 4). The intervention showed significant enhancements in motor efficiency, physical activity, and overall job satisfaction. Participants reported that the benefits were not only measurable but also deeply experienced, particularly in term of increased motivation, social support, and overall well-being.

**Table 4:** Parallelism between quantitative and qualitative analyses.

Feature	Quantitative results	Qualitative results (interview)	Comparison with the literature	Parallelism
Acquisition of motor efficiency and weekly physical activity	Motor efficiency improved significantly in the experimental group, with notable gains in cardiovascular fitness and flexibility. Weekly physical activity increased, particularly in light and moderate activities, with vigorous physical activity also rising significantly.	Participants reported physical improvements and greater awareness of the importance of regular physical activity. They expressed a desire for more opportunities for physical activity both within and outside the workplace. Some participants noted that the program encouraged them to increase their physical activity outside work hours, suggesting that the promotion of physical literacy in the workplace extended to their lifestyles outside of the office.	These findings align with the work of Rossi et al. (2025), who emphasized the positive impact of workplace interventions that integrate movement and promote physical activity. Their research showed that such interventions not only enhance cardiovascular fitness but also encourage employees to maintain a physically active lifestyle beyond work. Similarly, Walker et al. (2017) highlighted that physical activity interventions in the workplace, including active breaks and ergonomic improvements, reduce musculoskeletal pain caused by prolonged sitting, supporting the observed improvements in flexibility and motor efficiency in this study. Jindo et al. (2019) found that promoting physical activity in the workplace leads to sustained behavior changes, particularly in light and moderate-intensity activities, which were also observed in this study.	The quantitative improvements in physical activity and motor efficiency were mirrored in the qualitative feedback from participants, confirming that the intervention not only increased physical activity but also motivated employees to adopt healthier lifestyles outside the office. This corresponds well with the literature on the transfer of physical activity behaviors beyond the workplace environment.
Acquisition of psychological Well-being	The intervention led to an increase in overall psychological well-being, particularly in areas such as positivity, general health, and vitality.	Participants reported improved psychological well-being, with increased positivity and greater energy throughout the workday. Many also acknowledged that physical activity during work contributed to stress reduction and improved their mood.	The results align with Pronk (2014), who emphasized the positive connection between physical activity and mental health, noting that regular physical activity can reduce stress and enhance mood, which was evident in this study. Biddle et al. (2003) also highlighted that physical activity is closely linked to psychological well-being, and their findings were mirrored by the improvements in vitality and general health observed in the experimental group. The methodology employed in this study, using self-determination theory and need-supportive communication, is consistent with Ryan and Deci (2000), who argued that promoting autonomy, competence, and relatedness enhances intrinsic motivation and overall well-being. Participants in this study reported feeling more autonomous in managing their health, which aligns with these theoretical perspectives.	The quantitative improvements in psychological well-being were supported by the interviews, where participants noted that both physical activity and the need-supportive communication had a positive impact on their mood and overall well-being. These findings reinforce the literature on the role of physical activity in reducing stress and promoting emotional health.
Role ambiguity and job pressure	While direct measures of role ambiguity or job pressure were not included, improvements in psychological well-being and reduced job demands suggest that the intervention alleviated some of these challenges.	Participants described an initial period of confusion and role ambiguity, as well as excessive workloads due to limited resources. However, they also reported that, over time, these issues were mitigated through support from human resources and the integration of physical activities that helped reduce stress.	The issues of role ambiguity and work overload identified by participants are consistent with the findings of Vanishree (2014), who observed that such factors can negatively affect employee satisfaction and performance. However, the present study’s results suggest that integrating physical activity in the workplace, as recommended by Rossi et al. (2025), can help mitigate these challenges by promoting both physical and mental well-being. Hallam et al. (2022) found that promoting physical activity reduces work-related stress, which supports the qualitative findings in this study, where participants noted that the physical breaks reduced the psychological pressure associated with their roles.	The interviews highlighted the reduction in perceived role ambiguity and job pressure as a result of the intervention. While the quantitative data did not directly address these issues, the overall improvements in psychological well-being and social support align with the literature, which links physical activity interventions to reduced stress and improved role clarity.
Social support and job satisfaction	Participants showed increased perceived social support in the workplace, with a decrease in job demands, which was linked to reduced physical and psychological work stress.	Participants appreciated the support they received from colleagues and wellness coaches. They highlighted how active breaks and mutual support during work hours contributed to improved relationships and motivation.	The results align with Ghesmaty Sangachin et al. (2016), who emphasized the critical role of workplace support in achieving high levels of job satisfaction and wellness. This study supports the idea that perceived social support contributes to better employee outcomes, which was reflected in both the quantitative data and the qualitative feedback from participants. Jolly et al. (2020) found that social support during working hours can lead to greater motivation and well-being, a finding supported by the increased social support and job satisfaction reported in this study.	The quantitative findings of increased social support were mirrored in the participants’ interviews, where they reported feeling more supported by their colleagues and wellness coaches. This aligns with the literature, which underscores the importance of social support in fostering job satisfaction and well-being.
Positive effects after the two-years intervention	Follow-up results showed that the positive effects from the intervention were retained over time, with minimal dropout (just one participant in the experimental group). Statistical interactions revealed stable improvements in physical activity, suggesting that the intervention had lasting effects.	Participants expressed satisfaction with the continued support provided by wellness coaches and the lasting impact of the program. They highlighted how the continuous presence and education sessions helped them maintain the positive effects of the intervention.	The retention of behavior change observed in this study is consistent with findings from Clarkson et al. (2022), who argued that technological tools and continuous support play a crucial role in maintaining long-term behavior change. The integration of technology and wellness coaching likely contributed to the sustained effects of the intervention. The results also align with Invernizzi et al. (2021), who showed that the use of gamification and technology can enhance engagement and adherence to physical activity programs, leading to lasting behavior changes.	The retention of positive effects in this study, both quantitatively and qualitatively, aligns with the literature on the importance of ongoing support and technology in maintaining long-term behavior change. Participants reported that the continuous presence of wellness coaches and educational support helped them sustain the improvements in physical activity and overall well-being.

## 5 Limitations and perspectives

This longitudinal study was conducted within a single company, which limits the generalizability of the results to other workplaces or contexts. Further research is needed to evaluate how the UP150 concept can adapt to different work environments. Additionally, more data is necessary to examine the types of physical activity outside of work and how these may have been influenced during the trial. While the initial results are promising, additional research is required to fully understand the long-term impacts of these workplace interventions. Future studies should investigate the scalability of the UP150 concept by exploring its adaptation across various work environments, cultural contexts, and employee demographics. Not all organizations have the same resources or infrastructure to implement a comprehensive wellness program; therefore, it is essential to understand how to tailor it to meet diverse organizational needs.

Another important area for future research is evaluating the cost-effectiveness of workplace wellness programs. While there is increasing evidence that physical activity enhances employee health and productivity, it is crucial to demonstrate the return on investment (ROI) for companies that implement these programs. By quantifying the impact of wellness initiatives on absenteeism, healthcare costs, and overall productivity, organizations can make more informed decisions regarding their value.

Lastly, as technology evolves, digital health tools will play a more significant role in the future of workplace wellness programs. Future research should explore how developments in wearables, mobile apps, and other digital platforms can improve employee engagement, provide more accurate health data, and deliver personalized interventions. Understanding the evolving roles of wellness coaches, technology, and peer support will be essential in making the UP150 concept a lasting element of workplace culture.

## 6 Conclusion

The UP150 concept, implemented over a two-year trial, was associated with improvements in motor efficiency, physical activity levels, psychological well-being, and work-related well-being compared to the control group. The intervention appears to have encouraged employees’ physical activity during the workday, with positive effects on both physical and mental health. The involvement of wellness coaches and the use of technology supported employee engagement with the program, contributing to a more structured environment for health-promoting behaviors. Even if these findings are encouraging, they should be interpreted with caution, given the single-company study setting and the relatively small sample size, which limit generalizability. UP150 represents a potentially useful approach to addressing sedentary behavior and promoting activity in the workplace, but further research in diverse organizational contexts is needed to confirm these effects and explore long-term outcomes. Embedding wellness into organizational culture and leveraging technology may support ongoing behavioral change; additional studies are recommended to establish best practices for sustainable implementation.

## Data Availability

The raw data supporting the conclusions of this article will be made available by the authors, without undue reservation.
